# Exon skipping induced by nonsense/frameshift mutations in DMD gene results in Becker muscular dystrophy

**DOI:** 10.1007/s00439-019-02107-4

**Published:** 2020-01-09

**Authors:** Mariko Okubo, Satoru Noguchi, Shinichiro Hayashi, Harumasa Nakamura, Hirofumi Komaki, Masafumi Matsuo, Ichizo Nishino

**Affiliations:** 1 Department of Neuromuscular Research, National Institute of Neuroscience, National Center of Neurology and Psychiatry, 4-1-1 Ogawa-Higashi, Kodaira, Tokyo 187-8502 Japan; 2 Department of Pediatrics, Graduate School of Medicine and Faculty of Medicine, The University of Tokyo, Tokyo, Japan; 3 Department of Promoting Clinical Trial and Translational Medicine, Translational Medical Center, National Center of Neurology and Psychiatry, Tokyo, Japan; 4 Translational Medical Center, National Center of Neurology and Psychiatry, Tokyo, Japan; 5 KNC Department of Nucleic Acid Drug Discovery, Faculty of Rehabilitation, Kobe Gakuin University, Hyogo, Japan

## Abstract

Duchenne muscular dystrophy (DMD) is caused by a nonsense or frameshift mutation in the *DMD* gene, while its milder form, Becker muscular dystrophy (BMD) is caused by an in-frame deletion/duplication or a missense mutation. Interestingly, however, some patients with a nonsense mutation exhibit BMD phenotype, which is mostly attributed to the skipping of the exon containing the nonsense mutation, resulting in in-frame deletion. This study aims to find BMD cases with nonsense/frameshift mutations in *DMD* and to investigate the exon skipping rate of those nonsense/frameshift mutations. We searched for BMD cases with nonsense/frameshift mutations in *DMD* in the Japanese Registry of Muscular Dystrophy. For each *DMD* mutation identified, we constructed minigene plasmids containing one exon with/without a mutation and its flanking intronic sequence. We then introduced them into HeLa cells and measured the skipping rate of transcripts of the minigene by RT-qPCR. We found 363 cases with a nonsense/frameshift mutation in *DMD* gene from a total of 1497 dystrophinopathy cases in the registry. Among them, 14 had BMD phenotype. Exon skipping rates were well correlated with presence or absence of dystrophin, suggesting that 5% exon skipping rate is critical for the presence of dystrophin in the sarcolemma, leading to milder phenotypes. Accurate quantification of the skipping rate is important in understanding the exact functions of the nonsense/frameshift mutations in *DMD* and for interpreting the phenotypes of the BMD patients.

## Introduction

Dystrophinopathy is a group of progressive neuromuscular diseases caused by mutations in *DMD* (OMIM 300,377) located at Xp21.2. In male patients, a nonsense or frameshift mutation in the *DMD* gene cause a severe phenotype, Duchenne muscular dystrophy (DMD; OMIM 310,200), while in-frame deletion/duplication or missense mutation result in a milder phenotype, Becker muscular dystrophy (BMD; OMIM 300,376) (Monaco et al. 1988). Interestingly, however, some patients with a nonsense mutation or out-of-frame deletion/duplication of certain exons have been reported to show BMD phenotypes. In former cases, BMD phenotype is mostly attributed to the skipping of the exon containing the nonsense mutation, resulting in in-frame deletion (Shiga et al. 1997; Tuffery-Giraud et al. 2005), while in latter cases, variable splicing was caused around the genomic deletion, leading to restore the in-frame products (Anthony et al. 2014; Beggs et al. 1991; Deburgrave et al 2007; Kesari et al. 2008; Sherratt et al. 1993; Winnard et al. 1993; Tuffery-Giraud et al. 2017). For six nonsense mutations, the mechanism has been hypothesized as the point mutations resulting in either the critical disruption of exonic splicing enhancer (ESE) or creation of exonic splicing suppressor (ESS) motifs (Shiga et al. 1997; Disset et al. 2006; Kevin et al. 2011; Nishida et al. 2011). Furthermore, it also has been proposed that the effects of these single point mutations are dependent on weak intrinsic exon definition elements (Kevin et al. 2011). These exon skipping events by nonsense mutations are not specific to skeletal muscle, and they will be responsible for milder phenotype of not only skeletal muscle but also other organs in the patients. The minigene assay could be performed in HeLa cells as in previous reports (Nishida et al. 2011; Zhu et al. 2019). To understand these events and to simply evaluate the nonsense/frameshift mutations on the skipping rate of the corresponding exon, we analyzed exon skipping caused by the nonsense/frameshift mutations from a Japanese large cohort in artificial minigene experiments (Okubo et al. 2016, 2017).

## Methods

### Registry-based datasets

The Japanese Registry of Muscular Dystrophy (Remudy) was developed in 2009 in collaboration with the Translational Research in Europe-Assessment and Treatment of Neuromuscular Diseases (TREAT-NMD) Network of Excellence (Gatta et al. 2005; Lalic et al. 2005; Todorova et al. 2008). The Remudy database for male patients with dystrophinopathy includes clinical and molecular genetic data, as well as all mandatory and highly encouraged items for the TREAT-NMD global patient registry (Nakamura et al. 2013). All data were collected using the registrant’s self-report after obtaining the physician’s confirmation. Patient phenotypes were classified into three subgroups, DMD, intermediate muscular dystrophy (IMD), and BMD, based on the age at loss of ambulation [DMD < 13 years, 13 years ≤ IMD ≤ 16 years, and 16 years < BMD (Tuffery-Giraud et al. 2009)] and the results of dystrophin immunostaining in muscles. Genetic curators then independently evaluated the genetic data (Okubo et al. 2016). Pathological data (including dystrophin immunostaining, where applicable) were reviewed by clinical and genetic curators independently. In the present study, we used the registry data compiled from July 2009 to March 2017.

### Isolation of RNA from muscle biopsy and analysis of DMD transcripts

Total RNA was extracted from the muscle of patients by RNeasy mini kit (QIAGEN, Valencia, CA). Reverse transcription reaction was performed as previously described (Nishida et al. 2011). Primers used for amplification of dystrophin mRNAs are shown in Supplementary Table S1. PCR products were analyzed on 2% agarose gels in Tris–borate/EDTA buffer.

### Plasmid construction

The fragments encompassing exon and flanking intronic regions from nine exons were amplified by PCR. The primer designs are shown in Supplementary Table S2. We constructed minigenes with wild-type fragment of each exon into H492-dys plasmid (Nishida et al. 2011; Zhu et al. 2019). Mutations were introduced by Quick Change site-directed mutagenesis kit (Agilent Technologies, Palo Alto, CA, USA).

### Transfection and isolation of RNA

Transfection of the plasmids into HeLa cells was carried out using Lipofectamine 2000 (ThermoFisher Scientific, Waltham, MA, USA). At 24 h after transfection, RNAs were prepared by RNeasy mini kit (QIAGEN, Valencia, CA). Transfection was performed three times for each mutation and wild type.

### Quantitative real-time RT-PCR

Real-time RT-PCR amplification was performed as described previously (Nishida et al. 2011). It was performed one time for all RNA products from transfected HeLa cells. PCR products were separated by acrylamide gel electrophoresis and the intensity of each fragment was measured by 1D gel analysis of imageQuant TL (GE Healthcare, USA). Skipping rate was calculated as the intensity of the skipped fragment relative to sum of the skipped and unskipped fragments for each product (We have three products for each mutation). Then it was averaged, and the ratio of wild type was subtracted from each result of mutation for normalization.

### Splice site motifs, exon definition metrics

In silico analysis was performed by SpliceAid2 (https://193.206.120.249/splicing_tissue.html) (PIva et al. 2011) and Human Splicing Finder (HSF) (https://www.umd.be/HSF/) tool (Desmet et al. 2009; Kevin et al. 2011).

## Results

### Clinical characteristics

**Fig. 1:**
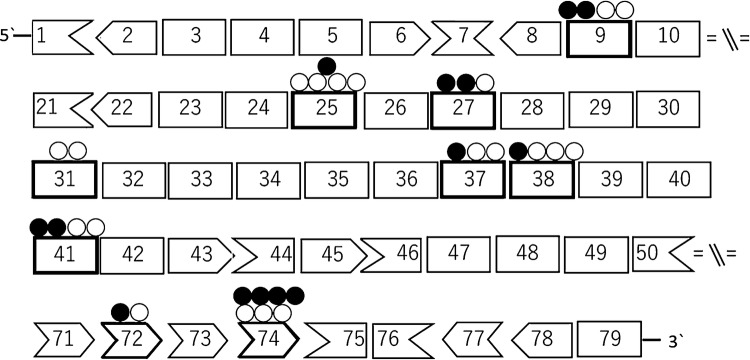
Nonsense/frameshift mutations in *DMD* gene identified in Japanese cohort. A schema representing exons of *DMD* gene and their reading frame by shape. White circles show position of nonsense/frameshift mutations identified in the patients showing positive dystrophin staining in their muscles. Black circles show positions in the patients with negative dystrophin staining

Among 1497 dystrophinopathy patients in our cohort, we found 18 cases with positive dystrophin staining from 363 cases with a nonsense or frameshift mutation in *DMD* gene. These 18 cases had 18 distinct mutations: 15 nonsense, a small indel, a 2-bp deletion and a 1-bp duplication which were located in exons 9, 25, 27, 31, 37, 38, 41, 72 and 74 (Fig. 1). For comparison, we also included 16 DMD patients with nonsense or frameshift mutations in these exons whose dystrophin was confirmed to be absent on muscle biopsy. Table 1 summarizes the clinical information of the 34 (17 BMD, 4 IMD, and 13 DMD) cases included in this study. Of note, the definitive phenotype of case no. 29, whose dystrophin was expressed on immunohistochemistry, could not be judged, as he is currently 11 years old and still ambulant albeit clinician’s impression was DMD. In cases no. 18, 27, 30 and 34, although their phenotypes were BMD or IMD, dystrophin staining was reported to be negative in the Remudy database. However, the muscle samples were not available, so we could not confirm their dystrophin staining.

**Table 1:** Summary of clinical features of patients

	Phenotype	Dystrophin IHC	Age	Ambulant	Age at loss of ambulation
1	DMD	Negative	8	y	
2	DMD	Negative	16	n	10
3	BMD	Positive	27	y	
4	BMD	Positive	12	y	
5	BMD	Positive	19	y	
6	BMD	Positive	8	y	
7	BMD	Positive	31	y	
8	DMD	Negative	24	n	9
9	BMD	Positive	56	n	54
10	DMD	Negative	9	y	
11	DMD	Negative	23	n	11
12	BMD	Positive	9	y	
13	BMD	Positive	14	y	
14	BMD	Positive	38	y	
15	IMD	Negative	14	y	
16	DMD	Negative	22	n	11
17	BMD	Positive	58	n	41
18	IMD	Negative	16	y	
19	BMD	Positive	23	y	
20	BMD	Positive	12	y	
21	DMD	Negative	5	y	
22	DMD	Negative	7	y	
23	DMD	Negative	9	y	
24	DMD	Positive	16	n	10
25	IMD	Positive	12	y	
26	BMD	Positive	43	y	
27	BMD	Negative	42	n	18
28	BMD	Positive	7	y	
29	DMD	Positive	11	y	
30	BMD	Negative	37	n	17
31	DMD	Negative	6	y	
32	DMD	Negative	5	y	
33	BMD	Positive	18	y	
34	IMD	Negative	14	y	

### Transcripts in biopsied muscle

**Fig. 2:**
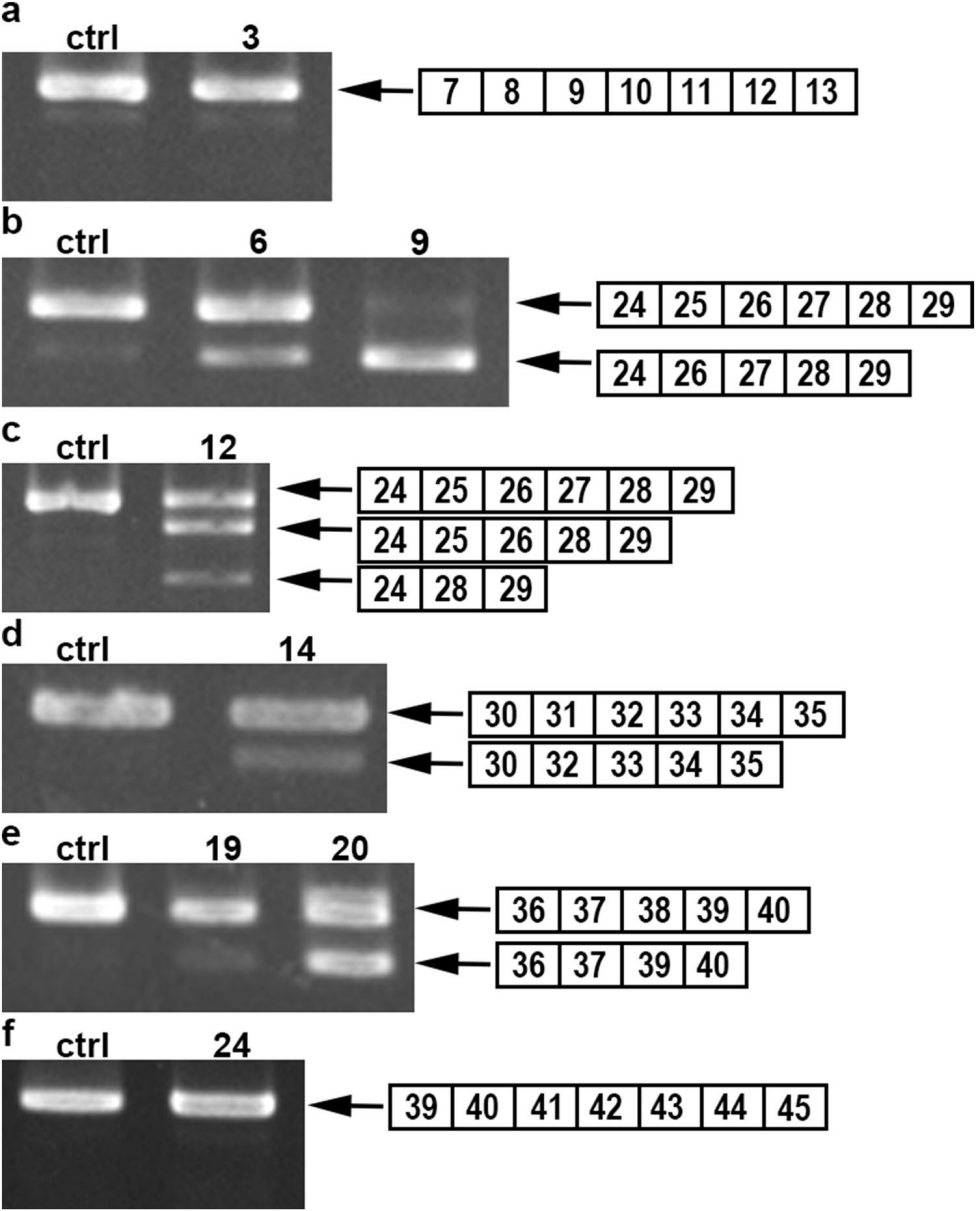
Exon skipping in DMD transcript from the patients with nonsense/frameshift mutations. The RT-PCR products obtained from the control and patients’ muscles are separated on agarose gels. The structure of each PCR product is shown schematically at the right of the panel. The RT-PCR products from the control and some patients showed only a single product (normal sequence) (**a**, **f**), while those from the other patients displayed additional shorter products (**b**–**e**)

Among 34 cases included in this study, frozen muscle samples were available in ten cases (Table 2). Two PCR products were found in six cases and three products in one case (Fig. 2b–e). Sequencing analysis revealed that the smaller products did not contain the mutated exon, suggesting the cause of exon skipping. However, only unskipped products were obtained in three cases (Fig. 2a, f).

**Table 2:** The results of RT-PCR from muscles

No.	Exon	Mutation	Amino acid change (predicted)	Exon skip in mRNA
3	9	c.883C > T	p.Arg295*	−
6	25	c.3304C > T	p.Gln1102*	**+**
7	25	c.3337C > T	p.Gln1113*	**+**
9	25	c.3430C > T	p.Gln1144*	**+**
12	27	c.3613G > T	p.Glu1205*	**+**
14	31	c.4327C > T	p.Gln1443*	**+**
19	38	c.5371C > T	p.Gln1791*	**+**
20	38	c.5407C > T	p.Gln1803*	**+**
24	41	c.5899C > T	p.Arg1967*	−
25	41	c.5899C > T	p.Arg1967*	−

### Skipping rate

**Fig. 3:**
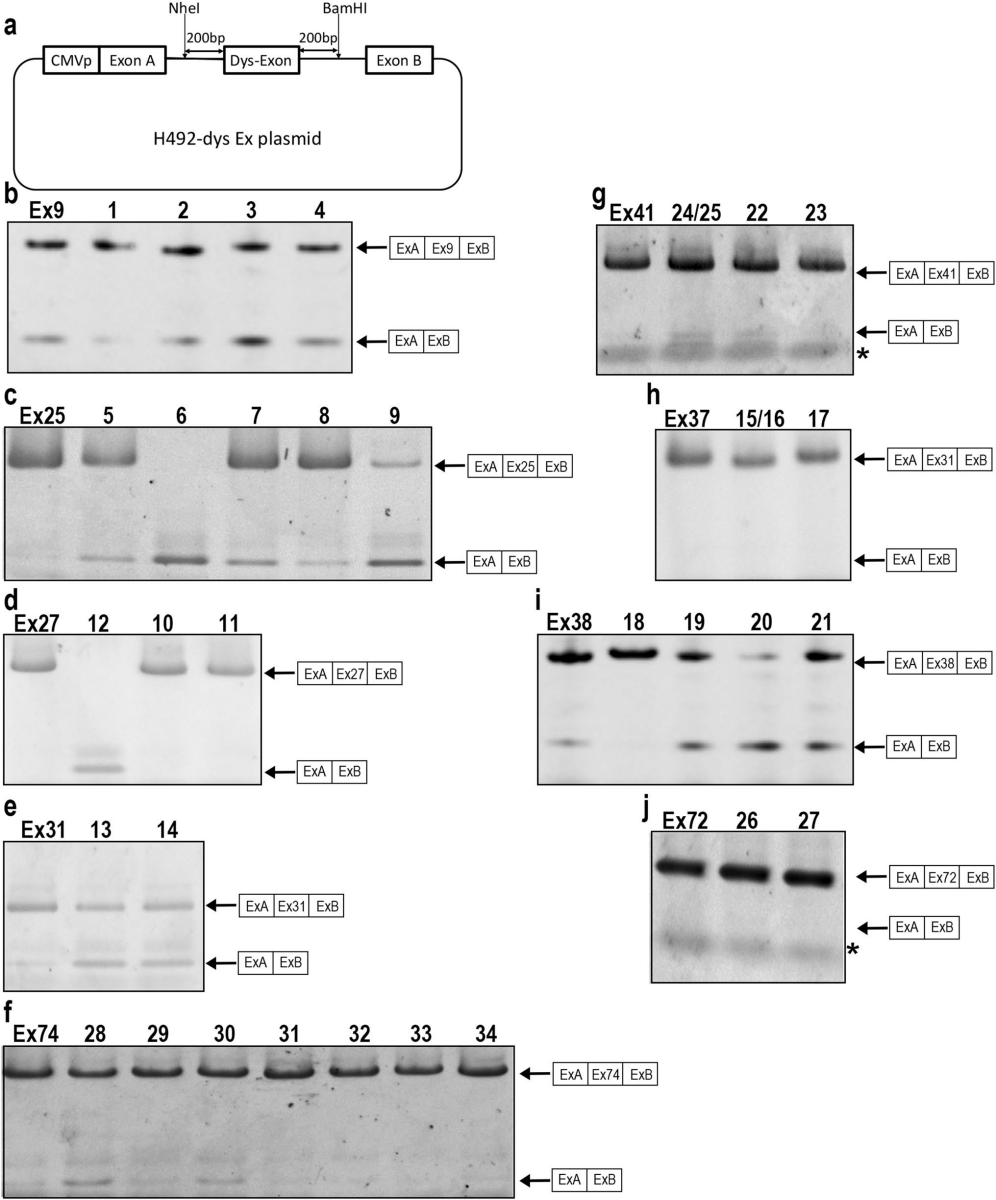
Nonsense/frameshift mutations caused exon skipping by H492-dys plasmid. **a** Schematic representation of H492 minigene constructs consisting of *DMD* gene region encompassing exon harboring a mutation (Dys-Exon) with flanking introns (200 bp each) and two artificial hybrid exons (exon A and B). **b**–**j** The RT-PCR products from HeLa cells transfected with wild-type or mutant H492 minigene were separated on agarose gel. The structures of each PCR product are shown at the right of the panels. The longer PCR products were included inserted exon and smaller one did not contain them without any cryptic splicing. *Primer dimer

Two RT-PCR products were amplified from some cells transfected with mutant plasmids, while only a single product was amplified from cells with the wild-type plasmid (Fig. 3b–j). Sequencing analysis revealed that the longer PCR products contained the inserted exon and smaller PCR products contain them exactly without any cryptic splicing. Calculated skipping rate is shown in Table 3. We found higher skipping rates from 0.05 to 0.98 in the nonsense/frameshift mutations were associated with BMD phenotypes, while those were from 0 to 0.03 in the mutations were associated with DMD phenotypes (Table 3). Among 34 cases we examined, 14 mutations caused exon skipping at a skipping rate of more than 0.05, while 18 mutations did not cause it at less than 0.05. Exon skipping rates were well correlated with dystrophin presence/absence and clinical phenotypes, suggesting exon skipping at 5% must be critical for the presence of dystrophin in sarcolemma and milder phenotypes. No exon skipping was observed in three mutations among the BMD cases (no. 17, 26, and 33). In five cases, there was a discrepancy between clinical phenotypes and dystrophin staining in muscles (no. 18, 27, 29, 30, 34), suggesting that dystrophin expression cannot explain the clinical phenotypes or the information of dystrophin expression by physicians might be misjudged. Without these five cases, exon skipping occurrence/non-occurrence was precisely observed in 89% of mutations (24/27).

**Table 3:** Summary of skipping rate in minigene experiments

No.	Exon	Mutation	Amino acid change (predicted)	Phenotype	Dystrophin IHC	Skipping rate in minigene
1	9	c.853G > T	p.Gly25*	DMD	Negative	0
2	9	c.857_858delAT	p.Tyr286*	DMD	Negative	0
3	9	c.883C > T	p.Arg295*	**BMD**	**Positive**	**0.15**
4	9	c.899_903CCTAC > TCTAG	p.Ala300_Tyr301delinsVal*	**BMD**	**Positive**	**0.08**
5	25	c.3295C > T	p.Gln1099*	**BMD**	**Positive**	**0.12**
6	25	c.3304C > T	p.Gln1102*	**BMD**	**Positive**	**0.98**
7	25	c.3337C > T	p.Gln1113*	**BMD**	**Positive**	**0.14**
8	25	c.3408_3409delinsGT	p.Gln1137*	DMD	Negative	0.03
9	25	c.3430C > T	p.Gln1144*	**BMD**	**Positive**	**0.73**
10	27	c.3734_3743delACTACCA	p.Asn1246Serfs*7	DMD	Negative	0.01
11	27	c.3765dupT	p.Gly1256Trpfs*15	DMD	Negative	0.03
12	27	c.3613G > T	p.Glu1205*	**BMD**	**Positive**	**0.65**
13	31	c.4294C > T	p.Gln1432*	**BMD**	**Positive**	**0.34**
14	31	c.4327C > T	p.Gln1443*	**BMD**	**Positive**	**0.3**
15, 16	37	c.5285delA	p.His1762Leufs*19	**IMD/**DMD	Negative	0.02
17	37	c.5287C > T	p.Arg1763*	**BMD**	**Positive**	0
18	38	c.5364dupA	p.Asp1789Argfs*7	**IMD**	Negative	0
19	38	c.5371C > T	p.Gln1791*	**BMD**	**Positive**	**0.09**
20	38	c.5407C > T	p.Gln1803*	**BMD**	**Positive**	**0.21**
21	38	c.5413dupG	p.Val1805Glyfs*10	DMD	Negative	0.04
22	41	c.5758C > T	p.Gln1920*	DMD	Negative	0.01
23	41	c.5770G > T	p.Glu1924*	DMD	Negative	0.01
24, 25	41	c.5899C > T	p.Arg1967*	**IMD/**DMD	**Positive**	**0.13**
26	72	c.10279C > T	p.Gln3427*	**BMD**	**Positive**	0
27	72	c.10320 T > A	p.Tyr3440*	**BMD**	Negative	0
28	74	c.10402G > T	p.Glu3468*	**BMD**	**Positive**	**0.12**
29	74	c.10406dupA	p.His3469Glnfs*22	DMD	**Positive**	0
30	74	c.10453_10454delCT	p.Leu3485Glufs*5	**BMD**	Negative	**0.05**
31	74	c.10454delT	p.Leu3485Argfs*11	DMD	Negative	0.01
32	74	c.10495G > T	p.Glu3499*	DMD	Negative	0
33	74	c.10498_10499delAG	p.Ser3500*	**BMD**	**Positive**	0
34	74	c.10500_10501delTG	p.Ser3500Argfs*14	**IMD**	Negative	0

Bold characters represent BMD phenotype, positive dystrophin staining, skipping ratio > 0.05

### Splice site motifs, exon definition metrics

By in silico prediction, both destruction of ESE and creations of ESS were predicted in 8 of the 14 mutations. In the remaining six mutations, only ESE destruction was predicted in two mutations, while only ESS creation was predicted in three mutations (Table 4 and Fig. S1).

**Table 4:** Summary of prediction of skipping events by SpliceAid2/HSF

No.	Exon	Mutation	Skipping rate in minigene	ESE disruption	ESS creation	Other motif
3	9	c.883C > T	**0.15**	−	**+**	
4	9	c.899_903CCTAC > TCTAG	**0.08**	−	**+**	
5	25	c.3295C > T	**0.12**	**+**	**+**	
6	25	c.3304C > T	**0.98**	**+**	**+**	
7	25	c.3337C > T	**0.14**	**+**	**+**	
9	25	c.3430C > T	**0.73**	**+**	**+**	Disrupt wt donor site
12	27	c.3613G > T	**0.65**	**+**	**+**	
13	31	c.4294C > T	**0.34**	**+**	**+**	
14	31	c.4327C > T	**0.3**	**+**	**+**	
19	38	c.5371C > T	**0.09**	−	**+**	
20	38	c.5407C > T	**0.21**	−	**+**	
24, 25	41	c.5899C > T	**0.13**	**+**	**+**	
28	74	c.10402G > T	**0.12**	**+**	−	
30	74	c.10453_10454delCT	**0.05**	**+**	−	

## Discussion

In this study, we found 14 BMD cases with a nonsense/frameshift mutation-mediated exon skipping, which accounts for 38.6% of 363 cases with a nonsense/frameshift mutation in *DMD* gene and 0.9% of 1497 dystrophinopathy cases in Japan. Albeit rare, it should be noted that there is a risk of misdiagnosis in those cases with such a genotype–phenotype discrepancy if the diagnosis was made solely based on the annotation of the mutations. Naturally, to make a precise diagnosis in such cases, not only genetic testing but also the combination of immunostaining, western blot, cDNA, in silico skipping prediction, and/or minigene analyses should be necessary.

This is the first systematic study to evaluate all *DMD* mutations which may cause exon skipping by in vitro experiments in the population. This report provides a large list of nonsense/frameshift mutations in *DMD* gene with exon skip causing rate and patients’ phenotypes. This catalogue could be useful as a reference for curative effects and will also help in further elucidating the nature of the disease.

Previous reports have identified 11 nonsense mutations associated with exon skipping, in exon 25 (Fajkusova et al. 2001; Santos et al. 2014; Zhu et al. 2019), in exon 27 (Shiga et al. 1997), in exon 29 (Ginjaar et al. 2000), in exon 31 (Disset et al. 2006; Nishida et al. 2011; Kevin et al. 2011), in exon 37 (Hamed et al. 2005), in exon 38 (Janssen et al. 2005; Kevin et al. 2011), and in exon 72 (Melis et al. 1998). In this study, we identified 11 nonsense and three frameshift mutations causing exon skipping in exon 9, 25, 27, 31, 37, 38, 41, 72 and 74, indicating the exon skip event is not restricted to nonsense mutations, but also due to other mutations. Nevertheless, all skipped exons were in-frame exons and single exon skipping was detected in all mutations.

As in the previous studies in which single exon skipping was evaluated by RT-PCR from muscle samples, we found single exon skipping in DMD transcripts from patients’ muscles except for the case of exon 27 (Fig. 2). In this study, we further characterized exon skipping events using H492-dys minigene plasmid that encompassed a single exon and flanking intronic regions. There are four advantages in experiments using the artificial minigenes. First, this method enables exon skipping measurement without patient samples. Second, it enables measurement of mutation effect based solely on exon skipping by removing confounding factors, i.e., intronic sequences and trans factors. Third, this method gives an accurate skipping rate without nonsense-mediated degradation of the unskipped transcript as compared to those found in skeletal muscle samples. The skipping rates based on RT-PCR products from skeletal muscles might be overestimated, since unskipped products with nonsense/frameshift undergo degradation by nonsense-mediated RNA decay. Fourth, the minigene assay can be conducted in non-muscle cell line such as Hela cells (Nishida et al. 2011; Zhu et al. 2019), because this nonsense-mediated exon skipping was not related to skeletal muscle-specific alternative splicing, but it will probably be observed in other organs. By this method, in fact, exon skipping occurrence/non-occurrence was reproducibly observed in 89% of mutations. Furthermore, an accurate skipping rate might be useful for predicting patient phenotypes and the results of clinical trials on exon skipping and readthrough.

All the previous reports on BMD with a nonsense mutation presented only the picture of the electrophoresed RT-PCR products as evidence of exon skipping, except for one report presenting sequencing data (Santos et al. 2014) and one in which quantitative data of each DMD transcript in the BMD patient were shown (Zhu et al. 2019). Therefore, the critical rate of exon skipping required for producing BMD phenotypes is still uncertain. In this study, we measured exon skipping rates in 32 mutations and found that when the exon was skipped at more than 5%, patients showed BMD phenotype. Of note, among the four mutations in exon 25, there was a negative correlation between skipping rate and phenotypic severity. In fact, with the mutation no. 9, the skipping rate was 0.73, and the patient could walk until age 54 years, while with the mutation no. 8, the skipping rate was 0.03 and the patient could not walk at age 9 years (Tables 1 and 3).

Our results, however, showed a discordance between the minigene assay and dystrophin immunostaining for mutations. Three mutations (no. 17, 26 and 33) did not induce exon skipping in minigene despite positive dystrophin staining in patients’ muscles and BMD phenotype. Exon 71–78 in *DMD* gene is known to be the region showing multiple exon splicing patterns in Dp71 (Austin et al. 1995). Nonsense mutations, no. 26 and 33, were located in exon 72 and 74, respectively. If such exons were alternatively spliced, it might be difficult to measure exon skipping rate in HeLa cells by this method. For the remaining mutation (no. 17) and those two mutations (no.26 and 33), we were unable to explain the discrepancy and we could not measure transcripts in muscle, as the muscle biopsy sample was not available.

In previous reports, the critical disruption of ESE or creation of ESS has been suggested to cause exon skipping (Shiga et al. 1997; Disset et al. 2006). By In silico prediction, among the 14 mutations which caused exon skipping, 10 mutations were predicted to disrupt ESE and 12 mutations were predicted to create ESS. From these results, it is strongly suggestive that both ESE disruption and ESS creation should be the mechanism for causing exon skipping. Thus, the in silico prediction is useful for interpreting results of minigene analysis.

Diagnosis is now based mainly on genetic testing and in many cases muscle biopsies are no longer carried out in patients with suspected dystrophinopathy. However, our study demonstrated the importance of biopsy to make a precise diagnosis. The multiple information on genetic tests, clinical information and pathological examination allowed both clinician and researcher to make an inclusive consideration for a prognosis.

## Data Availability

The datasets generated and analyzed during the current study are available in the Japanese Registry of Muscular Dystrophy (Remudy: https://www.remudy.jp/).

## Abbreviations

BMD: Becker muscular dystrophy
DMD: Duchenne muscular dystrophy
ESE: Exonic splicing enhancer
ESS: Exonic splicing silencer
HSF: Human Splicing Finder
IMD: Intermediate muscular dystrophy
Remudy: Registry of muscular dystrophy
